# An Unusual Case of Eosinophilia with Systemic Lupus Erythematosus: A Case Report and Review of Literature

**DOI:** 10.1155/2022/3264002

**Published:** 2022-02-28

**Authors:** Aman Mishra, Sandip Kuikel, Robin Rauniyar, Sagar Poudel, Sital Thapa, Nibesh Pathak, Suman Rimal, Kundan Raj Pandey, Saket Jha

**Affiliations:** ^1^Maharajgunj Medical Campus, Tribhuvan University Institute of Medicine, Kathmandu, Maharajgunj 44600, Nepal; ^2^Department of Internal Medicine, Tribhuvan University Institute of Medicine, Kathmandu, Maharajgunj 44600, Nepal; ^3^Rheumatology, Department of Internal Medicine, Tribhuvan University Institute of Medicine, Kathmandu, Maharajgunj 44600, Nepal

## Abstract

Eosinophilia can be caused by various conditions, parasitic infection being the most common cause. Here, we present a case of a 17-year male who presented with multisystem involvement and eosinophilia. He was later diagnosed to have systemic lupus erythematosus with eosinophilia which is a rare combination. Despite being a diagnostic challenge, these patients can be well managed with immunosuppressive therapy if recognized in time.

## 1. Introduction

Eosinophilia, defined as peripheral blood eosinophil count of more than 350 per mm^3^, is seen in many conditions [1]. Apart from parasitic infection which is the most common cause worldwide, allergy, hematological, and rheumatological conditions account for other etiologies [1, 2]. Although eosinophilia is commonly seen in rheumatological conditions such as eosinophilic granulomatosis with polyangiitis (EGPA) and less commonly in dermatomyositis, severe rheumatoid arthritis, progressive systemic sclerosis, and Sjögren syndrome, it has been rarely described in patients with systemic lupus erythematosus (SLE) [2, 3]. Here, we present a case of 17-year male who presented to our center with multisystem involvement and was diagnosed to have eosinophilic vasculitis with SLE.

## 2. Case Report

**Figure 1:**
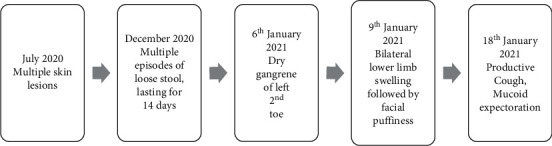
Timeline of symptoms onset.

A 17-year-male presented to our center with multiple skin lesions on bilateral lower limbs for 7 months. It started as painless, clear fluid-filled vesicles which later drained pus and resolved spontaneously. One month before the hospital presentation, he had 10–14 episodes of small-volume loose stool with mucus which lasted for 14 days. The diarrhea was not associated with fever, abdominal pain, and vomiting. Subsequently, he developed sharp, nonradiating pain at the tip of the left 2nd toe which progressed to dry gangrene involving half of the phalanx. He also complained of bilateral lower limb swelling and facial puffiness which subsided by the evening. He also had a productive cough with streaky hemoptysis without chest pain and shortness of breath. He also complained of fatigue, anorexia, and significant weight loss. There was no history of excessive hair fall, ear discharge, joint pain, malar rash, photosensitivity, oral and nasal ulcers, lumps and swelling, and muscle weakness. Patient did not have any comorbidities and has no history of any drug intake. A comprehensive timeline of his symptoms is shown in Figure 1.

**Figure 2:**
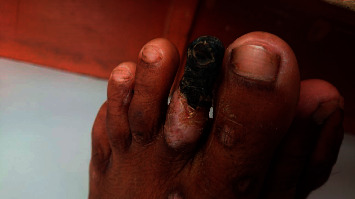
Dry gangrene in the left second toe.

**Figure 3:**
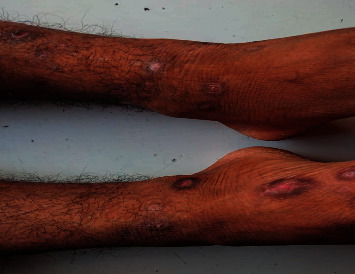
Multiple skin lesions with central hypopigmentation and perilesional hyperpigmentation.

On examination, pallor was present. Bilateral dorsalis pedis and left posterior tibial artery pulse were not palpable. Dry gangrene in the left second toe with a sharp line of demarcation was evident (Figure 2). Patchy loss of hair over bilateral toes was seen. Multiple skin lesions with central hypopigmentation and perilesional hyperpigmentation were seen on the bilateral lower limbs (Figure 3). There was diminished movement on the left side of the chest with a woody dull note on percussion and decreased vesicular breath sound.

The hemogram showed anemia (hemoglobin of 9.1 gm/dl) and eosinophilia (a total leucocyte count of 28560/mm^3^ with 36% eosinophils and an absolute eosinophil count of 10288/mm^3^). Inflammatory markers were raised (ESR 73 mm/hour and high sensitivity C-reactive protein 10680 ng/ml). His activated partial thromboplastin time (aPTT) was 37.7 s (control 29 s). On urinalysis, proteinuria was 1+ with a dipstick without active sediments, and 24-hour urine protein (UP) was 3.17 g/day. Peripheral blood smear showed increased corrected retics count and lactate dehydrogenase along with positive direct Coomb's test (Table 1).

Antinuclear antibody (ANA) tested positive with a homogeneous pattern and intensity of 3+ on immunofluorescence; however, his ANA immunoblot was negative. His dsDNA was raised, and antiphospholipid syndrome workup showed a borderline raised level of lupus anticoagulants and normal titres of anticardiolipin and anti-*β*2 glycoprotein I antibody. His C3 and C4 levels were normal (Table 2).

Contrast-enhanced computed axial tomography scan (CECT) of the chest and pulmonary angiography was done that revealed multiple intermediate-walled cavitary lesions in the bilateral lower lobes. A biopsy from the left lower lobe was done which revealed necrotic tissue. Other laboratory results did not show any signs of renal, electrolyte, or hepatic abnormalities. The Mantoux test and sputum for acid-fast bacilli yielded negative results. Tests for hepatitis A, B, and C, HIV1 and 2, and leptospirosis were negative. Repeated examination of blood, urine, sputum, and stool for bacterial and parasitic infection revealed no abnormalities. The stool revealed no RBC, parasite, egg, ova, or cyst.

He was started on high-dose oral prednisolone (1 mg/kg), mycophenolate mofetil, hydroxychloroquine, and anticoagulation. One week after the commencement of immunosuppressive therapy, 24-hour UP decreased to 0.29 g/day, and his hemogram revealed a decrease in eosinophil count to 13%. Three months later, on follow-up, the patient was symptom-free, and his absolute eosinophil count and 24-hour UP normalized to normal range.

## 3. Discussion

SLE is a multisystem autoimmune disorder with varying involvement of different organs [4].

In our case, there was the involvement of the vascular, hematological, respiratory, and renal systems. The investigation confirmed the diagnosis of SLE and persistent hypereosinophilia. Additional laboratory investigation was warranted to determine the cause of eosinophilia which included peripheral blood smear (for hematological malignancies), serological tests for hepatitis A, B, and C, HIV1 and 2, leptospirosis, blood, stool, and urine examination and culture. None of the tests were conclusive. There was no evidence for tuberculosis and aspergillosis as X-ray, CT scan, Mantoux test, and sputum for acid-fast bacilli yielded equivocal results. There was no evidence of parasitic infections. We planned to perform a tissue biopsy of the skin lesion and renal biopsy but could not be done as the biopsy specimen had to be sent to another country (for immunofluorescence), and it was unfeasible amidst COVID-19 nation-wide lockdown.

This manuscript not only points the unusual presentation of SLE with eosinophilia but also its presentation in a young male. SLE is more prevalent in female of child bearing age; however, it has various manifestations which vary according to the age group and gender because of difference in their pathogenetic mechanism [5]. Based on the history, clinical examination, and serum antibody positive for ANA, dsDNA, and lupus anticoagulant, we made a diagnosis of eosinophilic vasculitis with SLE with antiphospholipid antibody syndrome (APLAs). He can be diagnosed to have SLE on the basis of 2012 SLICC criteria as well as 2019 EULAR/ACR criterion. Positive ANA along with proteinuria >0.5 g/day with positive lupus anticoagulant and anti-ds DNA gives ≥10 with at least one clinical criterion point the diagnosis of SLE according to 2019 EULAR/ACR criteria for SLE. The diagnosis was further supported by a marked response to corticosteroid and MMF therapy. The main limitation and diagnostic challenge in our diagnosis was the unavailability of histopathological study of tissue biopsy of the skin lesions which would have guided our diagnosis of eosinophilic vasculitis earlier.

The other differential diagnoses to this presentation of this condition are parasitic infection, drug-induced lupus, and eosinophilic granulomatosis with polyangiitis. In this patient, serological tests and stool examination were carried out to exclude the presence of parasites, and no clinical signs of parasitic infection were present. Stool and serological tests were also negative for parasitic infection. The absence of intake of any drugs and negative antihistone antibody ruled out the possibility of drug-induced lupus. Although eosinophilic granulomatosis with polyangiitis is a possible diagnosis for this patient, negative P-ANCA and fulfilment of EULAR criteria make the diagnosis of SLE more favorable than eosinophilic granulomatosis with polyangiitis.

Increased serum LDH along with increased retics count in peripheral blood smear along with positive direct Coomb's test points that the cause of anemia in this case be autoimmune hemolytic anemia. The discordance between the proteinuria and anemia shall be nothing more than a mere coincidence. The presence of AIHA further adds to the positive correlation of this case to SLE.

Eosinophilia in the absence of coexisting allergy or parasitic infection is rarely seen in SLE patients. Eosinophilia with multiple organ/system involvement suggests eosinophilic granulomatosis with polyangiitis (Churg-Strauss vasculitis) as the primary working diagnosis. In the evaluation of such patients, it is recommended to test for toxocariasis, *Aspergillus* species (to evaluate for allergic bronchopulmonary aspergillosis), and human immunodeficiency virus. The laboratory investigations should include vitamin B12 level (to evaluate for myeloproliferative hypereosinophilic syndrome), peripheral blood smear (to look for dysplastic eosinophils or blasts suggestive of primary eosinophilic bone marrow process), and chest computed tomography scan (to evaluate for lung involvement) [2].

Several reports have described eosinophilic vasculitis [6], eosinophilic gastroenteritis [7, 8], Loffler's endocarditis [9, 10], eosinophilic pruritic cutaneous lesions [11], and nonerosive arthritis [12] in patients with systemic lupus erythematosus. All of the cases possessed diagnostic difficulty due to the rarity of the case. In most of the reported cases of SLE with eosinophilia, it was treated with high-dose corticosteroids and/or immunomodulators, and all cases responded well. In one case reported by Thomeer et al. [9], after the patient was clinically improved with the treatment, he died and a postmortem diagnosis of Loffler's syndrome was made. In another case report by Lee et al. [11], who used low-dose prednisolone (10–30 mg/day) for hypereosinophilic syndrome with SLE, there was no improvement in cutaneous manifestations. However, proteinuria in the patient had resolved. All the history, clinical, diagnosis, treatment, and result after treatment in reported cases of SLE with eosinophilia affecting different organs are given in Table 3.

This case report points to an atypical presentation of SLE with eosinophilia thus adding to the existing literature. The main limitation to this study is failure to obtain the skin and kidney biopsies, which could have confirmed the diagnosis in this patient. Due to the existing condition of worldwide pandemic, biopsy could not be done. However, the above discussed features support the diagnosis of SLE in this patient with atypical feature like eosinophilia.

## 4. Conclusion

We here present a rare association of SLE with eosinophilia. SLE should be kept in the differential diagnosis of persistent unexplained eosinophilia. Being a rare entity, diagnosis of this SLE with eosinophilia as a leading point can be difficult, but once diagnosed, this condition is responsive to high-dose corticosteroids and immunomodulator therapy.

## Figures and Tables

**Table 1:** Laboratory findings of the patient.

Parameters	Initial result at presentation	Results after 1 week of initial therapy	Results after 3 months of initial therapy	References
Urine				
Urine albumin	1+	Nil	Nil	
WBC	2–4	1–2	0–2	
24-hour urine protein	3.17 g/day	0.29 g/day	0.14 g/day	<0.15 g/day
24-hour urine creatinine	8200 *μ*mol/day	10997 *μ*mol/day	8000 *μ*mol/day	8840–13260 *μ*mol/day

Hemogram				
Total leucocyte count	28580/*μ*L	18840/*μ*L	6000/*μ*L	4000–11000/*μ*L
Hemoglobin	9.1 g%	8.5 g%	9.2%	13.5–17.5g%
Neutrophils	51%	57%	60%	
Eosinophils	36%	13%	2%	1–4%
Absolute eosinophils count	10288/*μ*L	8101/*μ*L	120/µL	50–500/*μ*L

**Table 2:** Serum antibody testing and its results of the patient.

Serum antibody	
Antinuclear antibody (ANA-IIF)	**Positive (1 : 320; 3+)**
Anti-ds DNA	**33.2 IU/ml (<30: negative)**
Complement 3	19 (reference range: 10–40)
Complement 4	122.2 (reference range: 90–180)
Anti-CCP	1.1 RV/ml (reference: <5.0 RV/ml)
hs-CRP	**10680 ng/ml (reference: 68-8200 ng/ml)**

ANA immunoblot	
Anti-Smith antibodies	Negative
U1 SM/RNP antibodies	Negative
SS-A antibodies	Negative
SS-B antibodies	Negative
RO-52 antibodies	Negative
Antihistone antibodies	Negative
Anticentromere antibodies	Negative

Rheumatoid factor	Negative
Anti-CCP Ab test	Negative

Lupus anticoagulant	**Positive**
Cardiolipin IgG and cardiolipin IgM	Negative
Anti-beta-2 glycoprotein 1 IgM and IgG	Negative

Anti-MPO antibodies	Negative
P-ANCA (IIF)	Negative
Anti-PR3 antibodies	Negative
C-ANCA (IIF)	Negative
Serum IgE	Normal
Direct Coombs test	**Positive**

Positive findings are denoted in bold.

**Table 3:** Clinical presentation, diagnosis, treatment, and outcome of different reported studies.

Author	Clinical presentation	Diagnosis	Treatment	Outcome
Hegarty et al. [6]	Fever, diarrhea, and vomiting along with a preceding history of fatigue and flitting polyarthralgia involving the wrists, hands, and feet	SLE, with hypereosinophilia, acalculous cholecystitis, and biopsy-proven eosinophilic vasculitis affecting the kidney	Three consecutive pulses of methylprednisolone (1 g/24 h)	Significant improvement
Asadi Gharabaghi et al. [7]	Five-month history of diarrhea and abdominal pain, scalp lesion similar to discoid lupus erythematosus.	Systemic lupus erythematosus with eosinophilic enteritis	Three daily pulses of methylprednisolone at a dose of 1,000 mg/day followed by 0.5 mg/kg/day prednisolone	Symptoms resolved
Jaimes-Hernandez et al. [8]	2 weeks of mild abdominal pain associated with nausea, vomiting, and melena	Eosinophilic enteritis with SLE	Exploratory laparotomy was done and later was diagnosed to be eosinophilic enteritis with SLE. Methylprednisolone pulses 1 g for three consecutive days	Symptoms resolved
Thomeer et al. [9]	Polyarthritis, pleuritis and pericarditis, alopecia, skin lesions, photosensitivity, anemia, lymphopenia, periungual vasculitis	SLE with eosinophilia with postmortem diagnosis of Loffler's syndrome	Methylprednisolone (40 mg/day), chloroquine (100 mg/day), and piroxicam (20 mg/day)	Death
Aydogdu et al. [10]	Confusion, quadriparesis, and ataxia	SLE and associated antiphospholipid syndrome with hypereosinophilia and Loffler's syndrome	1 mg/kg/day oral methylprednisolone, heparin followed by warfarin and chloroquine	Improved with further no thrombotic events
Lee et al. [11]	Malar eruption and extremely pruritic, lichenified papules, and plaques over the trunk and extremities for 2 months, followed by fever, myalgia, chest discomfort, and depressed mood Proteinuria	Hypereosinophilic syndrome associated with SLE	Prednisolone (60 mg/day) × 3 months Low-dose prednisolone (10–30 mg/day) × 1 year	Proteinuria-normal The eosinophil count was still high. Pruritus persisted and cutaneous lesion did not show improvement
Habibagahi et al. [12]	Dizziness, headache, and both upper and lower extremities weakness along with photosensitivity, malar rash, and livedo reticularis	SLE with hypereosinophilia with anticardiolipin antibody positive	Intravenous pulse methylprednisolone and therapeutic doses of heparin Maintained with 5 mg prednisolone every other day, warfarin (5 mg) and azathioprine (50 mg) per day as a steroid-sparing agent.	Improved

## Data Availability

The data used to support the findings of this study are available from the corresponding author upon request.
